# Multiple Roles of Glycerate Kinase—From Photorespiration to Gluconeogenesis, C_4_ Metabolism, and Plant Immunity

**DOI:** 10.3390/ijms25063258

**Published:** 2024-03-13

**Authors:** Leszek A. Kleczkowski, Abir U. Igamberdiev

**Affiliations:** 1 Department of Plant Physiology, Umeå Plant Science Centre, Umeå University, 901 87 Umeå, Sweden; 2 Department of Biology, Memorial University of Newfoundland, St. John’s, NL A1C 5S7, Canada; igamberdiev@mun.ca

**Keywords:** C_4_ photosynthesis, gluconeogenesis, glycerate metabolism, glycolate pathway, *Phytophthora infestans*, sucrose synthesis

## Abstract

Plant glycerate kinase (GK) was previously considered an exclusively chloroplastic enzyme of the glycolate pathway (photorespiration), and its sole predicted role was to return most of the glycolate-derived carbon (as glycerate) to the Calvin cycle. However, recent discovery of cytosolic GK revealed metabolic links for glycerate to other processes. Although GK was initially proposed as being solely regulated by substrate availability, subsequent discoveries of its redox regulation and the light involvement in the production of chloroplastic and cytosolic GK isoforms have indicated a more refined regulation of the pathways of glycerate conversion. Here, we re-evaluate the importance of GK and emphasize its multifaceted role in plants. Thus, GK can be a major player in several branches of primary metabolism, including the glycolate pathway, gluconeogenesis, glycolysis, and C_4_ metabolism. In addition, recently, the chloroplastic (but not cytosolic) GK isoform was implicated as part of a light-dependent plant immune response to pathogen attack. The origins of glycerate are also discussed here; it is produced in several cell compartments and undergoes huge fluctuations depending on light/dark conditions. The recent discovery of the vacuolar glycerate transporter adds yet another layer to our understanding of glycerate transport/metabolism and that of other two- and three-carbon metabolites.

## 1. Introduction

Plant GK is best known as the terminal enzyme of the glycolate pathway (photorespiration) [1,2]. It is also the last core enzyme of the glycolate pathway, which was genetically proven to be involved in photorespiration [3]. The pathway is the consequence of the oxygenase reaction of Rubisco, which produces 2-phosphoglycolate (2PG), a potent inhibitor of photosynthetic metabolism [4]. Aside from oxygenation, Rubisco is the primary carboxylation (CO_2_-fixing) engine in plants and is the key component of the Calvin–Benson–Bassham (CBB) cycle, responsible for biomass production. Rubisco’s oxygenation reaction is considered “unavoidable” under high [O_2_], as in the present-day atmosphere [5]. To remove 2PG from chloroplasts, plants evolved an elaborate pathway where 2PG-derived carbon is transported through several cell compartments, resulting in a net loss of one carbon per two molecules of 2PG (a total of four carbons) that are produced in the cycle. The remaining 75% of carbon is returned to chloroplasts in the form of glycerate, which is converted to 3-phosphoglycerate (3PGA) by GK. The 3PGA then re-enters the CBB cycle for subsequent reduction and sugar formation [1,2,6]. 

In this review/opinion paper, we argue that GK should not be considered merely as a concluding step of photorespiratory metabolism, but rather as showing functional flexibility. Evidence is discussed that shows that the photorespiratory flux can be diverted from the CBB cycle via a newly discovered cytosolic form of GK and directed either to the synthesis of sugars in the cytosol via biosynthetic reactions of reverse glycolysis (gluconeogenesis), or to the oxidative reactions of glycolysis and the tricarboxylic acid (TCA) cycle. Recent discoveries of the vacuolar glycerate transporter and the possible role of GK in C_4_ metabolism are also discussed. Finally, an unexpected link between GK and plant immune response is examined. It is concluded that the GK isoforms are involved in alternate pathways for the utilization of glycerate and that GK plays various essential roles in plant biology.

## 2. GK—Gene and Protein

Plant glycerate kinase (GK) carries out the following reaction: D-glycerate + ATP → D-3-phosphoglycerate (3PGA) + ADP. It is representative of a so-called class III GK, while classes I and II refer mostly to bacterial and animal GKs [7]. Besides plants, the class III GKs can also be found in some cyanobacteria such as Nostoc [7] and Anabaena [3], in yeast [8], and in some algae, e.g., *Cyanophora paradoxa* [9]. Cyanobacteria were the first organisms in which oxygenic photosynthesis evolved and, consequently, were the first in which the Rubisco oxygenase reaction had commenced [9,10]. Some algae, such as diatoms and green algae, lack genes for GK [11,12,13,14]. This is due to a peculiar C_2_ pathway in these organisms, where glycolate oxidation is carried out by peroxisomal glycolate dehydrogenase instead of glycolate oxidase, and glyoxylate thus formed is subsequently incorporated into malate and further metabolized in the TCA cycle (reviewed in [14,15]). 

Arabidopsis GK is encoded by a single nuclear gene (*GLYK*) [3,9] composed of 11 exons, the first one coding for the plastid transit peptide [3,7]. Plant GK differs from bacterial and animal GKs both structurally and functionally [7]. The most important functional difference is that plant GK produces 3PGA, which is also the first stable product of the CBB cycle, also known as the C_3_ cycle, whereas bacterial and animal GKs produce 2PGA [7,16,17,18,19]. 

There have been several reports describing the purification of native plant GK [3,20,21,22,23,24]. Purified recombinant GKs were also analyzed [3,7,25]. The purified enzyme is a monomer with a molecular mass of ca. 40 kD and uses D-glycerate and ATP as its substrates. The use of D-glycerate (but not L-glycerate) was found both for purified GKs from spinach and maize leaves and for crude leaf extracts [17,18], suggesting that leaves contain no kinase capable of the utilization of L-glycerate. For purified spinach GK, based on substrate kinetics and kinetics with its products and analogs, the enzyme reaction is consistent with a sequential random mechanism, where D-glycerate and ATP bind to the enzyme in a random fashion before the products (3PGA and ADP) are released [17].

Surprisingly, to our knowledge, no plant or animal GK has been crystallized and its structure solved. However, based on amino acid sequencing of plant GKs and comparisons to known structures of GK from yeast and non-plant microorganisms, several domains shared between plant and non-plant GK proteins have been identified [3,25].

## 3. GK Regulation and Subcellular Location

### 3.1. Transcriptional and Post-Transcriptional Regulation

Oxidative stress and intense light cause damage to the photosynthetic apparatus, especially PSII, resulting in photo-inhibition. Under such conditions, many photorespiratory enzymes are upregulated at both transcript and protein levels. This alleviates oxidative stress and contributes to photoprotection [26,27,28,29]. The GK gene (*GLYK*) is regulated at the transcriptional and post-transcriptional levels by light and metabolites [30,31,32]. Similar to several other core photorespiratory genes, the *GLYK* promoter contains an expression-enhancing 5′-untranslated intron, which is enriched in signals that elevate gene expression [31,33]. In wild-type Arabidopsis, light upregulation of *GLYK* expression was accompanied by approximately a two-fold increase in the leaf content of GK protein after the onset of light, which decreased after the light was switched off [30]. In some photorespiratory mutants, an increase in glycine and serine contents had stimulating effects on *GLYK* expression [30,34]. 

**Figure 1:**
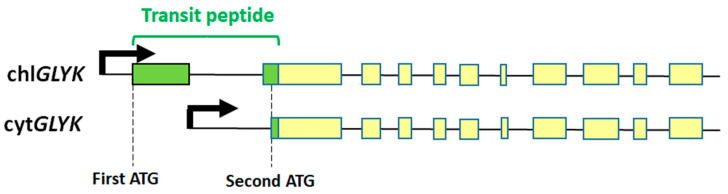
A simplified view of two pre-mRNAs produced from alternative promoters of a single Arabidopsis *GLYK* gene, coding for chloroplastic (chl*GLYK*) and cytosolic (cyt*GLYK*) isoforms of GK (modified from [35]). Boxes represent exons. Transit peptide coding sequence is shown in green. Positions of first and second ATGs in the *GLYK* coding sequence are also indicated. Light-dependent transcription of the gene is under phytochrome control and involves alternative promoter selection, resulting in two mRNAs coding for the chloroplastic and cytosolic isoforms of GK, respectively [35].

One of the highlights in recent studies on GK was the discovery of the phytochrome-dependent regulation of Arabidopsis *GLYK* expression, resulting in the production of two transcripts, coding for chloroplastic and cytosolic isoforms of GK, respectively [35] (Figure 1). Until then, GK had been generally considered as being located exclusively in plastids [21,36,37], although there were also hints of a cytosolic GK (see below). The appearance of the cytosolic isoform was correlated with the shading/darkening of the plants, and the expression of both isoforms was initiated by alternative promoter selection under phytochrome control [35,38]. This was later confirmed both for Arabidopsis GK and for GKs from several Solanaceae plants [39]. Aside from these GKs, we do not know whether the cytosolic GK is also present in other species (e.g., C_4_ plants) or in non-photosynthetic tissues. The full-length GK transcript (containing a transit peptide for chloroplast targeting) predominantly accumulated in 12 h light/12 h dark conditions, whereas its content was reduced upon continuous dark exposure. In contrast, the truncated transcript (coding for the cytosolic isoform) was upregulated in 24 h dark conditions [39]. The chloroplast and cytosol locations for GK proteins were conclusively confirmed by using constructs of *GLYK* with fluorescent markers expressed in transgenic plants, by analyses of the N-termini of both proteins, and with antibodies specific for each isoform [35,39]. 

The presence of a cytosolic GK was earlier reported for spinach leaves based on the non-aqueous fractionation of leaf tissue [40]. This cytosolic activity was about half of the GK activity in chloroplasts, and the same distribution was found in young and mature leaves. As an almost aside note in the early review on photorespiration, Heber and Krause [6] wrote in 1980: “Phosphorylation of glycerate is possible both inside and outside the chloroplasts”. These conclusions are now independently confirmed by two other laboratories [35,39]. 

### 3.2. Redox Regulation

Maize GK was the first enzyme of the glycolate pathway found to be “light-regulated”, with the regulation occurring via the chloroplastic thioredoxin system [41]. The maize enzyme was fully active only when extracted from illuminated leaves and had low activity when isolated from darkened leaves. The stimulating effect of light could be mimicked in crude extracts from darkened leaves by the addition of reduced thioredoxins or other reducing agents [41,42]. Interestingly, redox regulation was found only in GKs from the leaf extracts of certain C_4_ plants of the NADP-malic enzyme subtype (e.g., maize, sorghum, and sugarcane) and not C_3_ plants or C_3_–C_4_ intermediates [42]. 

Subsequent studies have demonstrated that maize GK has two redox-sensitive cysteines (Cys), separated only by a few amino acids at the very end of its C-terminal domain. In the night, these Cys residues undergo oxidation to form a disulfide bridge and make GK less active. However, in the light, the disulfide bridge is reduced by thioredoxin to restore full activity to the enzyme [25]. The two Cys residues are found only in GKs from C_4_ plants of the NADP-malic enzyme subtype, but not in all of them [25]. Fusion of the maize GK Cys-containing redox domain to the C-termini of GKs from C_3_ plants conferred redox regulation to these otherwise unregulated enzymes [25]. For GK from maize and some other C_4_ plants, this “light/dark” regulation, which could be also mimicked by incubation with reducing and oxidizing agents, resulted in over ten-fold changes in GK activity [25,42]. Despite the marked activation, treatment of recombinant maize GK with DTT and thioredoxin had no effect on *K*m values with its substrates (D-glycerate and ATP) [25]. Thus, the observed activation of GK was entirely due to changes in catalysis and not changes in the substrate affinity of the enzyme.

Potential redox regulation of GK, aside from some C_4_ plants, was also found for the enzyme from *Chlamydomonas reinhardtii*, which becomes nitrosylated after the cells are treated with *S*-nitrosoglutathione [43]. Whether the nitrosylation affects the activity of GK is unknown. GK is also regulated by persulfidation [31], but again it is unknown whether it affects GK activity. Apart from GK, several other enzymes of the glycolate pathway are prone to redox control [44].

### 3.3. Metabolite Regulation

Plant GK was competitively inhibited by its product 3PGA. In both C_3_ and C_4_ plants, the magnitude of in vivo GK activity could be a function of the ratio of stromal concentrations of ATP to 3PGA, and possibly to other phosphoesters (e.g., nucleoside bisphosphates) [17,24]. However, no major regulatory mechanisms at the metabolite control level were found. It has been suggested that the activity of GK from C_3_ plants is modulated solely by substrate availability [17,22]. This early view of an unregulated GK, barely working as a terminal part of the glycolate pathway machinery and delivering 3PGA to the photosynthetic carbon metabolism, was challenged by later findings of redox control of GK in C_4_ plants [25,41,42], especially the light-regulated presence of chloroplastic and cytosolic GK isoforms (see above). 

## 4. Origins of D-Glycerate

### 4.1. HPR (and GR) Isozymes/Isoforms

Plants have several genes coding for hydroxypyruvate reductase (HPR) [45,46] and glyoxylate reductase (GR) [47]. Out of these two enzymes, only HPRs can produce glycerate (from hydroxypyruvate); however, HPRs in most cases can also reduce glyoxylate (to glycolate) [48,49]. In cell extracts, the HPRs’ glyoxylate-dependent activities could easily be confused with the glyoxylate-dependent activities of true GRs, which do not react with hydroxypyruvate [50]. Glyoxylate is best known as a glycine precursor in photorespiration and as a Rubisco activase inhibitor, but it is also central to the metabolism of one-, two-, and three-carbon compounds [51,52]. Thus, we have decided to also include GRs here in our discussion of plant HPRs. 

Among HPRs, only HPR1 strongly prefers NADH as its cofactor, while HPR2 and HPR3 are more active with NADPH. This concerns both hydroxypyruvate- and glyoxylate-dependent activities [49,53]. Interestingly, Arabidopsis HPR3 is more active with glyoxylate than with hydroxypyruvate [53]. In single-celled Chlamydomonas, five genes have been identified coding for HPR activities [54].

HPR1 and HPR2 are located in the peroxisomes and cytosol, respectively, whereas HPR3 is likely based in the chloroplasts [46,53,55]. In pumpkin, the gene for HPR1 encodes two HPR1 isoforms (peroxisomal and cytosolic) that are produced via light-regulated alternative splicing of pre-mRNA [56,57]. The role of the cytosolic isoform is unknown, but it seems unlikely that it corresponds to the HPR2 protein since HPR1, HPR2, and HPR3 are coded by separate genes [45,46,53,58,59]. 

In Arabidopsis and rice, both HPR1 and HPR2 are involved in photorespiratory metabolism, as found by mutant analyses [45,53,60]. Analyses of Arabidopsis HPR3 knockouts also linked its phenotype to photorespiration [53]. Comparisons of Arabidopsis single, double, and triple (*hpr1*/*hpr2*/*hpr3*) mutants revealed that, among single mutants, the strongest photorespiratory phenotype belonged to *hpr1*, followed by *hpr2* and then *hpr3*. The triple mutant showed increased growth retardation and decreased photochemical efficiency compared to other double and single mutants [53]. Arabidopsis HPR mutants, especially *hpr1*, have been employed as useful tools in unraveling the details of metabolic linkages of photorespiration with plant primary metabolism [30,61].

Certain non-photosynthetic tissues, e.g., barley seed endosperm, contain little if any HPR1 activity, but have considerable HPR2 and/or HPR3 activity [62]. This suggests that HPR2 and/or HPR3 may also have functions other than in photorespiration, e.g., can be linked to serine formation from glycerate (via their reverse reactions) [63,64,65,66]. Another proposed role for HPR2 involvement, together with isocitrate dehydrogenase, is in cytosolic NADPH/NADP^+^ turnover. In barley seed endosperm, HPR2 could use carbon skeletons derived from abundant amino acids, including serine [62].

At the post-translational level, the HPR proteins are modified by persulfidation (HPR1 and HPR2), *S*-nitrosylation (HPR1), *S*-sulfenylation (HPR2) [31], phosphorylation (HPR1) [67], and tyrosine nitration (HPR1) [68]. Out of these modifications, only the last two were further investigated. In both cases, the activity of HPR1 was decreased, with the phosphorylation affecting the cofactor specificity of the enzyme [67], and with the nitrosylation implying a link between photorespiration and peroxisomal NO metabolism [68]. 

Apart from mutant approaches, glycerate metabolism can be studied using, more or less, specific inhibitors of enzymes that produce glycerate, most importantly peroxisomal HPR1 and cytosolic HPR2. These two can be distinguished from one another in crude plant extracts by (i) their high reactivity with NADH (HPR1) or NADPH (HPR2), (ii) differential fractionation with ammonium sulfate [69], and (iii) differential sensitivity to oxalate. Oxalate selectively inhibits HPR2 but not HPR1 activities [45,62,69,70,71,72] (Table 1). Purified spinach HPR2 has *K*i values with oxalate as low as 7 µM (with hydroxypyruvate and NADPH) and 36 µM (with glyoxylate and NADPH) [71]. Purified recombinant HPR3 was also inhibited by oxalate [53]. Thus, both HPR2 and HPR3 can be distinguished from HPR1 by their oxalate sensitivity. Interestingly, inhibition by oxalate was also reported for purified recombinant rice GR1 and GR2 isozymes, but their *K*i values with oxalate were 2–3 orders of magnitude higher than those for spinach HPR2, especially for NADPH-dependent activity [47]. Analyses of the ammonium sulfate-fractionated leaf extracts of the barley *hpr1* mutant revealed that HPR2/HPR3, but not GR1/GR2, were also inhibited by tartronate and phosphohydroxypyruvate (apparent *K*i values of ca. 0.3 and 0.4 mM, respectively) [59].

Concerning GR enzymes, based on analyses of tobacco suspension cells transiently transformed with Arabidopsis GRs genes, the proteins are localized to the cytosol (GR1) and plastids (GR2) [73,74]. A mitochondrial isozyme/isoform of GR was also reported [75]. Earlier studies on GRs, using fractionated leaf protoplasts, have also pointed to the cytosol and plastid locations of the two isozymes [55,76]. Studies on rice mutants revealed that both cytosolic GR1 and plastidial GR2 are simultaneously required under high photorespiratory conditions (low [CO_2_]) but are functionally redundant under normal growth conditions [47]. In contrast to C_3_ plants, maize leaves appear to contain very little, if any, GR1/GR2 activity [69,72]. 

Inhibitors found to affect GRs’ activities, but not those of HPRs, were acetohydroxamate, aminooxyacetate, and glycidate [69,70]. Out of these compounds, acetohydroxamate was found to be the most effective by far (*K*i of ca. 0.3 mM) in differentiating between GR1 and glyoxylate-dependent HRP activities [69,70].

Besides glycerate/glyoxylate metabolism, both HPR2 and GR1 can also be involved in other pathways due to their ability to react with alternative substrates. Thus, in addition to the production of D-glycerate from hydroxypyruvate, HPR2 can also utilize hydroxyphenylpyruvate as an alternative substrate to synthesize 4-hydroxyphenyllactic acid, a key precursor to rosmarinic acid (RA) [77,78]. RA is one of the most common caffeic acid esters and is especially prevalent in the Lamiaceae (e.g., *Coleus blumei*) and Boraginaceae (e.g., forget-me-not species) plant families. RA production rarely occurs outside of these plant families, and thus it is possible that HPR2 may serve as a vital link to RA formation in addition to its role in photorespiration. In addition, HPR2 was recently linked to Arabidopsis primary root growth in response to Pi deficiency [79]. The GR1 enzyme, on the other hand, in addition to reacting with glyoxylate, can also carry out the reaction of succinic semialdehyde reductase, where succinic semialdehyde (SSA) is reduced by NADPH to γ-hydroxybutyrate (GHB), which is the product of the metabolism of γ-aminobutyrate (GABA), a ubiquitous, non-protein amino acid involved in signaling and plant stress/defense responses. These non-specific activities of HPR2 and GR1 are of physiological importance [77,80], even though the purified enzymes strongly prefer to use hydroxypyruvate and glyoxylate, respectively, as their substrates [74,81]. Both HPR2 and GR1 may serve as examples of “moonlighting” enzymes, i.e., having different roles depending on the substrate available. They are involved in the photorespiratory pathway (HPR2 and, to some extent, GR1), and/or secondary metabolism (HPR2), and/or GABA catabolism (GR1).

The substrate specificities, subcellular locations, and selective inhibitors of HPRs and GRs are summarized in Table 1.

### 4.2. 3PGA Phosphatase

The cytosolic pool of D-glycerate is also under the control of 3PGA phosphatase activity. This enzyme, first characterized by Randall and Tolbert [82], has mostly eluded further studies. The enzyme from sugarcane, located in the cytosol of mesophyll cells, has been proposed to play a role in a pathway from D-glycerate to serine in C_4_ plants [82]. It has also been proposed that the enzyme has a role in the 3PGA/triose-P shuttle between the bundle sheath and mesophyll cells in C_4_ plants [42,83] (see below).

The 3PGA phosphatase produces D-glycerate from 3PGA as its preferred substrate and its activity is highest at low pH (optimum pH 5.9, characteristic for the cytosol), whereas it becomes unstable at a pH above 7.5. In sugarcane leaves, 3PGA phosphatase activity exhibits a diurnal variation, increasing during late daylight hours and early evening. In the light in C_4_ plants, this enzyme is usually more active than in C_3_ plants [82,83].

### 4.3. Lactate Dehydrogenase 

Another enzyme capable of producing glycerate from hydroxypyruvate is lactate dehydrogenase (LDH). However, the reaction produces only the L-stereoisomer of glycerate, which cannot be subsequently metabolized by GK (GK uses only D-glycerate as a substrate). LDH can also produce lactate from pyruvate [84,85]. 

### 4.4. Tartronic Semialdehyde Reductase 

Tartronic semialdehyde reductase (TSR) can produce D-glycerate from tartronic semialdehyde and is involved in bacterial glyoxylate metabolism. Plants apparently lack TSR activity, but cyanobacteria use TSR in one of at least three pathways that they employ to metabolize 2PG [10]. Bacterial TSR was frequently used in the production of transgenic plants with reduced photorespiration and enhanced yield (e.g., [86,87]). 

## 5. Glycerate Transporters

### 5.1. Plastidial Glycerate Transporters

The plastidial glycolate/glycerate transporter (PLGG1) transfers D-glycerate into chloroplasts in exchange for glycolate [88]. The transporter belongs to a unique group of metabolite carriers [89], and it is absolutely required for photorespiration [89,90]. In Arabidopsis, the transporter is encoded by a single gene and is located in the inner chloroplast membrane [89]. In contrast, rice contains two glycolate/glycerate transporters, located in the inner and outer chloroplast membranes, respectively, but interacting with each other as a singular complex. Both of these transporters are encoded by separate genes [91]. Chloroplasts also contain another glycolate translocator (BASS6), but it does not transfer glycerate, and thus only PLGG1 accounts for the glycerate import [92]. Activities of both BASS6 and PLGG1 are responsible for the observed stoichiometry of the two glycolate molecules exported from chloroplasts in exchange for one glycerate during photorespiration.

### 5.2. Vacuolar Glycerate Transporter 

The presence of the vacuolar D-glycerate transporter (VGT), located in the tonoplast of vacuoles, was initially deduced from metabolomics work, and further studies showed that a loss-of-function *vgt* mutant had reduced the content of glycerate in the vacuoles [93]. Based on gene analyses, the VGT protein contains six transmembrane domains [93], which is unusual among the hundreds of known transmembrane carriers, as they usually have 12 transmembrane domains [94]. Nevertheless, injection of VGT-coding mRNA into the oocytes of *Xenopus laevis* elevated the efflux of glycerate, proving that the transporter is indeed functional [93]. The mutant lacking VGT showed growth retardation and an early senescence phenotype upon nitrogen depletion. This and other evidence have suggested that glycerate transport into the vacuole alleviates the impact of an increased C/N ratio under N deficiency. Thus, photorespiration may regulate the C/N balance, in addition to scavenging carbon [93].

The vacuole is the fifth compartment involved in photorespiratory metabolism, in addition to chloroplasts, peroxisomes, mitochondria, and the cytosol [93,95]. Two alternative roles for the vacuolar transporter were proposed. Firstly, glycerate accumulated in the vacuole acts as a kick-start for glycolate export from chloroplasts at the onset of photorespiration. In this scheme, glycerate is exported from the vacuole to serve as a substrate for the chloroplastic glycerate transporter in exchange for glycolate export. The second role is that the VGT contributes to the expansion of photorespiratory bypasses, with glycerate joining cytosolic pools of serine, glyoxylate, hydroxypyruvate, and other small compounds derived from the glycolate pathway [95]. Aside from VGT, the key contributing activities to the cytosolic glycerate pool are HPR1, HPR2, and 3PGA phosphatase. This pool is controlled by cytosolic GK, which itself is under light (phytochrome) control [35].

## 6. GK in Photorespiration

The breakthrough in studies on GK came with the production of Arabidopsis T-DNA-induced mutants of GK that ultimately proved GK’s role as the core enzyme of the glycolate pathway [3]. Chloroplastic GK is thought to salvage part of the glycolate-derived carbon as 3PGA and deliver it to the CBB cycle. The mutants showed no GK activity and were not viable in normal air. However, they grew under elevated concentrations of CO_2_, similar to mutants of the other enzymes of the glycolate pathway [3]. Similar conclusions were drawn from more recent studies on Arabidopsis GK mutants generated by CRISP/Cas9 technology [96].

Upon diurnal growth of Arabidopsis, there is a huge increase (up to 30-fold) in glycerate content in the light compared to night conditions [30]. Given that the amount of GK protein increases only two-fold in the light, the increase in glycerate content during the light suggests that the in situ activity of GK is rather low and characterized by a high control coefficient [30]. This is surprising, since metabolic pathways that are far from equilibrium, as is the case with the glycolate pathway, are usually controlled by the activity of upstream enzymes [97]. However, although GK is certainly downstream in the glycolate pathway, it may also be considered as an upstream reaction in the cytosolic link of glycerate metabolism. Another possibility is that most glycerate is located in the vacuole in the light, away from general metabolism. The limiting rate of the chloroplastic GK and its regulation at the transcriptional and post-transcriptional levels by light and metabolites in C_3_ plants [30,31,32], and at the enzymatic level by thioredoxin [41], provide fine regulation of the entry of photorespiratory carbon to the CBB cycle. 

Whereas the chloroplastic isoform of GK retrieves the glycolate-derived carbon back to the CBB cycle, the cytosolic isoform appears to have more distinct roles. Depending on light conditions and phosphate supply, photorespiratory carbon can be metabolized in the cytosol, which could be preceded by glycerate accumulation in the vacuole via the glycerate transporter [93,95]. In the next section, we will discuss the importance of the cytosolic pathway of glycerate metabolism in maintaining the balance of carbon fixation, phosphate availability, and sucrose synthesis in changing environmental conditions. 

## 7. GK in Gluconeogenesis and Respiration 

**Figure 2:**
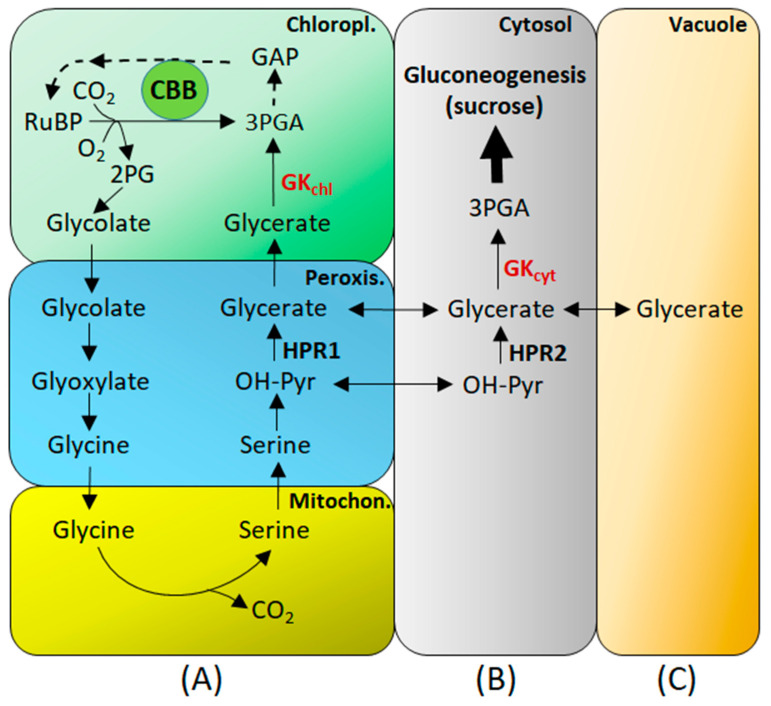
Glycerate metabolism and the diurnal roles of GK in primary metabolism (in C_3_ species). (**A**) The glycolate pathway during the day; (**B**) Cytoplasmic bypass of glycerate toward gluconeogenesis and glycolysis. (**C**) The role of the vacuole as a retainer for glycerate flux (day and night). Five compartments (marked with different colors) are involved. Abbreviations: CBB, Calvin–Benson–Bassham cycle; GAP, glyceraldehyde phosphate; GK_chl_, chloroplastic GK; GK_cyt_, cytosolic GK; HPR, hydroxypyruvate reductase; 2PG, 2-phosphoglycolate.

The two distinct isoforms of GK derived from the single gene [35] determine the alternate routes for the glycerate formed in the photorespiratory pathway. While the chloroplastic isoform of GK operates downstream in the glycolate pathway, the cytosolic isoform represents an upstream reaction for gluconeogenesis and glycolysis (Figure 2). 

The cytosolic GK isoform is part of a cytosolic bypass of the photorespiratory pathway, similar to that by HPR2 [49] or to the reported serine bypass, where as much as 23–41% of glycolate-derived serine is exported into other metabolic pathways [98]. The bypass allows redistribution of the glycolate-derived metabolites toward other pathways (Figure 2B). In this scheme, the product of cytosolic GK (3PGA) can enter gluconeogenesis and glycolysis in the cytosol rather than the CBB cycle in the chloroplasts. This provides a direct link between glycolate pathway metabolism and the primary pathways in the cytosol. 

It has been suggested that the appearance of the cytosolic GK in shaded plants helps them to adjust metabolically to fluctuating light conditions [35,38]. In photorespiratory conditions, the direction of the photorespiratory flux of glycerate to sucrose via gluconeogenesis would be preferable over its oxidation in glycolysis since the latter would result in the additional loss of the fixed carbon. The reductant and ATP are generated in mitochondria during oxidation of the photorespiratory glycine, while respiration is suppressed in the light [99,100,101]. Sucrose synthesis via gluconeogenesis, on the contrary, keeps carbon in the cell and balances Pi concentration. However, cytosolic GK can contribute to glycolysis under shade/darkness. The fact that glycerate content is much larger in the light, when compared to dark conditions [29], may reflect shade/dark-dependent increase in the content of cytosolic GK isoform.

From 3PGA as a starting point, sucrose synthesis is entirely cytosol-located, while the reactions of glycolysis are present in both the cytosol and plastids, but the most intensive gluconeogenetic/glycolytic flux is in the cytosol [102,103,104]. The reduction power for cytosolic 3PGA reduction to triose-P may come both from chloroplasts and mitochondria [105]. In the light, chloroplast redox shuttles are very active, and the mitochondrial glycine oxidation also provides the reductant exceeding the NADH-dissipating capacity of mitochondria [106]. Since the cytosolic glycerate conversion is supposed to intensify under plant shading/darkening [35,39], in these conditions, light inhibition of respiration is alleviated, and light-enhanced dark respiration provides an additional amount of reductant [107]. Thus, the respiratory NAD(P)H can be used to convert 3PGA to triose phosphates. The source of ATP for the GK reaction in the cytosol is also primarily mitochondrial since chloroplast membrane is not easily penetrable for ATP, while the active transport of adenylates via the mitochondrial membrane provides a supply of energy by mitochondria to the cytosolic anabolic reactions [108,109].

The existence of cytosolic GK suggests that the glycolate pathway could be also regarded as an elaborate means of exporting 2PG-derived carbon to the cytosol and converting it, via several reactions, to cytosolic 3PGA for e.g., sucrose formation (gluconeogenesis) (Figure 2B). On the way, a variety of distinct two- and three-carbon compounds, including glycine and serine, are produced and utilized in further metabolism. Another possibility is glycerate retention in the vacuole, depending on leaf metabolic status (Figure 2C). Glycerate, the GK substrate, emerges as an important link between photorespiratory metabolism and other primary pathways, and the cytosolic isoform of GK must be considered as a yet another contribution of the cytosol proteome to the bypasses of photorespiratory metabolism. Other examples are HPR2 and GR1 [55,74,76,110].

Cytosolic GK could provide clear benefit to the efficiency of photosynthesis. In a modification of the then existing model of carboxylation under Pi-limiting conditions, it has been proposed that if fraction of the carbon lost to the chloroplast as glycolate fails to return to the chloroplast as glycerate, this will result in a net increase in stromal Pi [111]. In that case, phosphate normally used by chloroplastic GK to form 3PGA would become available for photophosphorylation, stimulating RuBP regeneration and CO_2_ assimilation. Phosphate depletion in chloroplasts occurs, in particular, when starch and sucrose synthesis fails to match the capacity for the production of triose-P in the CBB cycle. To invoke the net release of phosphate, a part of the glycolate carbon must remain in the cytosol [111]. Glycerate formed from the glycolate carbon should not necessarily be stored in the vacuole or converted to amino acids, but could use the cytosolic pool of phosphate to be converted to sucrose via gluconeogenesis, followed by re-entry of phosphate from the cytosol to the chloroplast. It is known that under Pi limiting conditions, the pools of photorespiratory metabolites (glycolate and glycine + serine) are markedly increased, and the synthesis of soluble sugars from photorespiratory intermediates is enhanced [112].

The optimal rate of photosynthesis occurs when the phosphate concentration is low enough to allow starch and sucrose synthesis at the required rate and high enough for ATP synthesis at the required rate [113]. Fine mechanisms of regulation of the chloroplastic and cytosolic GK could result in the flexibility in balancing phosphate concentration in photosynthetic cells to provide optimal matching between photophosphorylation and sucrose synthesis in the conditions of changing such parameters as light intensity and the internal CO_2_ concentration, which increase leads to Pi depletion [114]. Thus, the concentration of phosphate that is necessary to regulate photosynthesis can be under control via the involvement of the chloroplastic and cytosolic forms of GK. Increased sucrose synthesis, facilitated by increased content of glucose-6-phosphate, an activator of sucrose-phosphate synthase, would recycle Pi from the cytosol back to the chloroplast, maintaining ATP, RuBP and hence net photosynthetic rate [115].

## 8. GK in C_4_ Metabolism

**Figure 3:**
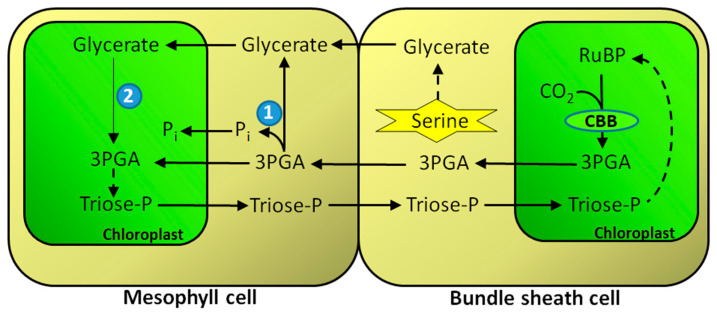
Flow of glycerate from the bundle sheath cells to the mesophyll cells during C_4_ photosynthesis. (1) 3PGA phosphatase; (2) Glycerate kinase. Abbreviations: CBB cycle, Calvin–Benson–Bassham cycle; Pi, inorganic phosphate.

Even though photorespiration does not occur or is severely reduced in C_4_ plants, due to CO_2_ concentrating mechanism there [116,117], these plants have full set of enzymes similar to those involved in photorespiration in C_3_ species, but having different metabolic functions. A case in point can be made for GK in C_4_ plants, especially that it is the only enzyme of the “classical” glycolate pathway that is exclusively localized in mesophyll cells, away from bundle sheath cells where Rubisco is localized. The obvious source of glycerate used by GK comes from bundle sheath cells metabolism, where glycerate can be formed from serine via combined reactions of serine-glyoxylate aminotransferase (SGAT) and HPRs [37,118] (Figure 3). In the conditions of suppressed photorespiration, the alternate source of serine could be so called phosphorylated serine pathway, which releases phosphate in the chloroplast and thus stimulates photosynthesis [66,119,120]. SGAT in plants can be encoded by more than one gene (two in *Lotus japonicus*—[121]), although most likely it is located exclusively in peroxisomes and possesses also serine: pyruvate aminotransferase and asparagine: glyoxylate aminotransferase activities [122]. In C_4_ plants, bundle sheath cells have higher activity of SGAT and HPRs than mesophyll cells, and their activities, especially HPRs, are comparable to those in C_3_ plants [69,123]. Another possibility for origin of glycerate is the involvement of 3PGA phosphatase. 

In C_4_ plants, 3PGA phosphatase is located predominantly in the cytosol of mesophyll cells and its activity is generally higher than in leaves of C_3_ species [83]. In C_4_ plants, both GK and 3PGA phosphatase can be involved in the flow of 3PGA from the bundle sheath cells to the mesophyll in exchange for the flow of triose-P in the reverse direction. The flow of 3PGA can be described as facilitative diffusion due to the involvement of mesophyll 3PGA phosphatase, which increases the gradient of 3PGA between the bundle sheath and mesophyll cells (Figure 3) [42,83]. 

Glycerate is transported into mesophyll chloroplasts by the glycerate/glycolate translocator (as in C_3_ plants) [124]. This, coupled with GK activity in the stroma, results in the formation of 3PGA, which is converted to triose-P. Many C_4_ plants, especially of the NADP-malic enzyme type (e.g., maize), divide the reduction of 3PGA to triose-P between the CBB cycle in the bundle sheath chloroplasts and the mesophyll chloroplasts [125,126]. Since mesophyll cells do not contain Rubisco [127], 3PGA synthesized by GK in mesophyll chloroplasts forms a distinct pool from 3PGA produced by Rubisco in bundle sheath cell chloroplasts. However, in plants with the NADP-malic enzyme as a primary decarboxylation mechanism, the chloroplasts in bundle sheath cells are characterized by a substantial deficiency of photosystem II activity (no thylakoid stacking), resulting in a limited production of NADPH which is required for the reduction of 3PGA to triose phosphates [128,129,130]. Thus, as much as one-half of the 3PGA formed in these chloroplasts has to be exported to the mesophyll chloroplasts for reduction, where it eventually forms a common pool with 3PGA derived from glycerate (by GK). Mesophyll chloroplasts have normally developed thylakoid grana and show high rates of both ATP and NADPH production during the light phase of photosynthesis [131]. 

As carbon flow between the bundle sheath cells and the mesophyll occurs predominantly by diffusion via plasmodesmata [116,132,133], this requires the building up of concentration gradients of 3PGA (high concentration in the bundle sheath cells) and triose-P (high concentration in the mesophyll), as indeed is the case [134]. In this way, the mesophyll-derived triose-P contributes to the buildup of CBB cycle intermediates in the bundle sheath cells. Metabolite fluxes in C_4_ photosynthesis are believed to represent modified remnants of metabolic shuttles distributing photorespiratory metabolites between the mesophyll and bundle sheath cells [135,136,137,138]. The movement of glycerate (along with 3PGA) between the bundle sheath and mesophyll cells might be a part of such an old shuttle. 

The reason why GK from some C_4_ plants is light/thiol-regulated [25,41] is still unclear. Since C_4_ plants evolved from C_3_ species [135], it seems possible that the capacity for light activation of GK arose in response to the increased need for an effective flow of 3PGA and triose-P necessary to sustain photosynthesis during C_4_ metabolism. It has been suggested [25,139] that the excess of glycerate produced in the light by 3PGA phosphatase activity can be stored in the vacuole. Early next morning, the glycerate could be exported from the vacuole and used by light-activated GK to rapidly produce 3PGA for the accelerated synthesis of triose-P in the mesophyll and its export to the bundle sheath cells to maintain the CBB cycle [134,140]. The light activation of GK is very rapid, with maize leaf GK increasing its activity several-fold within a few minutes [41]. However, as pointed out by Bauwe [139], the existence of such a mechanism in C_4_ plants has not yet been verified, the key question being whether the vacuolar glycerate retention, as demonstrated for C_3_ plants, exists in C_4_ species. 

GK is likely to play a role in sucrose synthesis in C_4_ species. In these plants, sucrose is formed almost entirely in mesophyll cells, whereas starch is confined to the bundle sheath cells [141,142,143]. GK may be an essential player in C_4_ plants as a link between CO_2_ fixation in bundle sheath cells and sucrose synthesis in the mesophyll cytosol. 

Finally, recent metabolomics studies of various C_3_, C_4_, and C_3_–C_4_ intermediate species of Flaveria revealed an unexpectedly large glycerate pool in C_4_-type Flaveria in comparison to C_3_–C_4_ and C_3_ Flaveria species [144]. It has been suggested that glycerate acts as a carbon reserve that can be used to replenish the levels of CBB cycle metabolites [144]. A large buildup of glycerate requires enzymes capable of producing it in large amounts (e.g., HPRs), but also a storage space, possibly a vacuole.

## 9. GK and Plant Immunity

It is well known that photorespiration plays important roles in defense responses, and recent evidence highlights the importance of chloroplast-generated reactive oxygen species production in response to pathogen infection [145]. For instance, levels of hydrogen peroxide, a key signaling molecule in plant immunity, are strongly impacted by photorespiration [146,147]. Photorespiratory metabolites, the interaction between photorespiration and defense hormone biosynthesis, and other mechanisms are also implicated [148,149].

**Figure 4:**
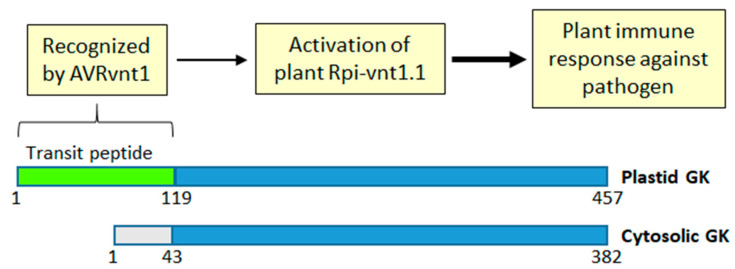
A cartoon-like comparison of amino acid (aa) sequences of potato GK chloroplastic and cytosolic isoforms. From aa #119 (chloroplastic GK) and aa #43 (cytosolic GK), the proteins share 100% identity. The proteins are produced by a light-dependent *GLYK* promoter selection mechanism (see Figure 1). The chloroplastic GK, but not cytosolic GK, contains a transit peptide (in green) which directs the protein to chloroplasts, and is removed upon the transfer. The transit peptide is recognized by AVRvnt1, an effector protein produced by *Phytophthora infestans*. This triggers plant protein Rpi-vnt1.1, which is involved in plant immune defense.

Recently, it has been reported that GK is involved in conferring immunity to potato plants against *Phytophthora infestans*, a potent pathogen of plants of the Solanaceae family. It was the full amino acid sequence (including the transit peptide) of chloroplastic GK (produced in the light), but not the transit-peptide-less cytosolic isoform (produced in the dark) (see Figure 1), that was essential for conferring GK-assisted immunity against the pathogen [39]. The transit peptide of the chloroplastic GK was essential for binding AVRnt1, the effector protein secreted by Phytophthora, which then led to activation of the specific plant protein, Rpi-vnt1.1, involved in the light-dependent immune response [39] (Figure 4). Interestingly, although transit peptides of GK from Solanaceae plants (potato, tomato, and *Nicotiana benthamiana*) interacted with AVRvnt1, the transit peptide of Arabidopsis GK failed to do so. This was likely due to the observed divergence of the amino acid sequence of the Arabidopsis GK transit peptide when compared to those of GK from the other plants [39]. It has been proposed that Rpi-vnt1.1 is activated when GK trafficking to chloroplasts is interrupted by AVRvnt1. The Rpi-vnt1.1 either senses the GK-AVRvnt1 complex or responds to changes in GK conformation caused by binding of the effector. Alternatively, Rpi-vnt1.1 senses the GK transit peptide that is released upon transfer of GK to the chloroplasts [39].

## 10. Summary and Perspectives 

The glycolate pathway initially evolved to prevent suicide by photosynthesizing cells when atmospheric O_2_ concentration increased (due to O_2_ evolution during photosynthesis) and Rubisco oxygenase activity started producing toxic 2PG [139,150]. This happened long before the appearance of higher plants in the early Proterozoic period at the time of the Great Oxidation Event [151,152]. All organisms performing oxygenic photosynthesis require the glycolate pathway to survive, because even low amounts of 2PG, a product of the Rubisco oxygenase reaction, are deadly for the cell (reviewed in [139]). Before developing the glycolate pathway metabolism as we know it now for C_3_ plants (see Figure 2A), the mechanisms to eliminate the toxic effects of 2PG varied, e.g., cyanobacteria have three pathways for the metabolism of 2PG [10]. Later on, upon the emergence of higher plants, the “classical” glycolate pathway of C_3_ plants was developed. Still later, parts of the glycolate pathway’s basic machinery gave rise to C_3_-C_4_ and C_4_ metabolism and provided a variety of small compounds, which feed other major pathways, e.g., gluconeogenesis and glycolysis. In this respect, the recently uncovered light-dependent dual cytosol-chloroplast localization of GK [35,39] (Figure 1), although so far shown only for GK from C_3_ species, may play an important role. In fact, the light-dependent changes in the ratio of chloroplastic to cytosolic GK isoforms [35,39] may have fundamental effects on the observed flexibility of photorespiratory metabolism in fluctuating light conditions [153]. 

**Figure 5:**
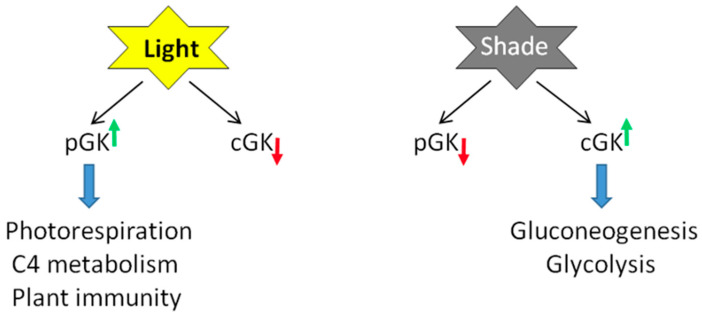
Consequences of light regulation of the GK gene on protein amount/activity of GK isoforms. Green and red arrows refer to an increase or decrease of GK protein/activity, respectively. The light/shade (or light/dark) regulation of the GK promotor affects the ratio of the chloroplastic to cytosolic GK isoforms, the former prevalent in the light, and the latter building up in shade/night conditions. pGK, plastidial GK; cGK, cytosolic GK.

Figure 5 summarizes light’s involvement in the production of both isoforms of GK. With glycerate shown as a major metabolite and its contents strongly fluctuating in light/dark conditions [30], together with the discovery of a glycerate transporter in the vacuole [93,95], the finding of cytosolic GK constitutes a whole new game-changer with respect to links between photorespiration and other pathways. GK, instead of being a rather unremarkable last enzyme of the pathway, comes out as one of major players. Having said so, nothing is yet known about the enzymatic properties of cytosolic GK. Its amino acid sequence differs only at the N-terminal domain from that of already characterized chloroplastic GKs (Figure 4). The N-domain may contain some amino acids interacting with/stabilizing the binding of ATP, the GK substrate, as found in the crystal structure of a GK-like yeast protein [25,154]. Thus, the cytosolic and chloroplastic isoforms may differ in their affinity for ATP. 

It is unclear whether C_4_ plants, similar to C_3_ species, contain an isoform of cytosolic GK in addition to the chloroplastic one in the mesophyll cells (Figure 3). This would have provided an obvious shortcut to sucrose synthesis and respiration in this tissue. The same uncertainty concerns the presence of the glycerate carrier in vacuoles of C_4_ plants, either in bundle sheath cells, mesophyll cells, or both cell types. Vacuoles could provide retention and distribution centers for glycerate (Figure 2C).

More studies are needed on the effects of inhibitors on glycerate-producing enzymes. To our knowledge, no metabolomics studies were performed with plants treated with any of the HPR and GR inhibitors (Table 1). Obviously, the key question would be whether these compounds are specific to their intended target(s) [155,156]. The effects of oxalate would be most interesting, as it does not interfere with peroxisomal HPR1, whereas it is extremely effective, at least in some plants, in inhibiting cytosolic HPR2 [62,71,72]. Thus, it can be used at low concentrations in such studies. Glyoxylate, rather than ascorbate, is considered as the main source of oxalate [157] in the side reaction catalyzed by glycolate oxidase. Since peroxisomes represent the major source of glyoxylate, the lack of inhibition of HPR1 by oxalate guarantees the sustained flow of the photorespiratory pathway in peroxisomes, while cytosolic hydroxypyruvate conversion is highly controlled. Another advantage of using oxalate is that it acts as a typical uncompetitive inhibitor of HPR2 [62,71]. When used in vivo, uncompetitive inhibitors are unlikely to be replaced from the active/regulatory sites on the enzyme by an increasing the concentration of its substrates [158,159]. Alternatively, the inhibitors could be used in crude plant preparations for quick discrimination between HPR1, HPR2/HPR3, and GR activities. As both HPR2 and GR1 can use alternative substrates not related to the glycolate pathway (Table 1), these non-specific reactions can also be studied, both in vivo and in crude extracts, with the same inhibitors. 

Finally, the involvement of GK in immune recognition in plants (Figure 4) is quite unexpected, but in hindsight, it may be linked to GK involvement in photorespiration and, subsequently, to the eminent role of this pathway in plant immunity responses [148]. Certainly, more studies are needed to further probe this exciting link between GK and plant immunity.

The multiple roles of GK outlined above suggest that the enzyme can be considered the proverbial jack of all trades in plant biology. GK is involved in several pathways of primary metabolism, but also in plant immune responses. Its subcellular location in the chloroplasts and/or cytosol is light-dependent and overall GK activity depends on a diurnal cycle. Glycerate, the GK substrate, also emerges as an important organic compound, trafficking between different plant compartments and linking photorespiratory metabolism with other primary pathways. Cytosolic GK makes the reversion of photorespiratory flux from the CBB cycle possible, linking it to the synthesis of sucrose in the cytosol through gluconeogenesis and to the oxidative reactions of glycolysis. The function of leaf (photorespiratory) peroxisomes thus becomes more similar to the function of glyoxysomes. Whereas glyoxysomes link flavin-dependent β-oxidation of fatty acids to gluconeogenesis [160,161], leaf peroxisomes may provide the linkage of flavin-dependent oxidation of a short-chain acid (glycolate) to gluconeogenesis [35], in addition to sugar-phosphate metabolism in the CBB cycle.

## Figures and Tables

**Table 1:** Substrate specificity, localization, and selective inhibitors of HPRs and GRs *. Other enzymes producing glycerate are also shown.

Enzyme	Reaction	Location	Inhibitors
HPR1	OH-pyruvate + NAD(P)H → D-Glycerate + NAD(P)^+^ Glyoxylate + NAD(P)H → Glycolate + NAD(P)^+^	Per	None
HPR2	OH-pyruvate + NAD(P)H → D-Glycerate + NAD(P)^+^ Glyoxylate + NAD(P)H → Glycolate + NAD(P)^+^ OH-phenylpyruvate + NAD(P)H → 4-OH-phenyllactic acid + NAD(P)^+^	Cyt	Oxalate, Phosphohydroxypyruvate, Tartronate
HPR3	OH-pyruvate + NAD(P)H → D-Glycerate + NAD(P)^+^ Glyoxylate + NAD(P)H → Glycolate + NAD(P)^+^	Chl	Oxalate
GR1	Glyoxylate + NAD(P)H → Glycolate + NAD(P)^+^ SSA + NAD(P)H → γ-OH-butyrate + NAD(P)^+^	Cyt	AHA, AOA, Glycidate, Oxalate?
GR2	Glyoxylate + NAD(P)H → Glycolate + NAD(P)^+^	Chl, Mit	Oxalate?
PGA-Pase	3PGA → D-Glycerate + Pi	Cyt	None
LDH	Pyruvate + NAD(P)H ⇌ Lactate + NAD(P)^+^ OH-pyruvate + NAD(P)H ⇌ L-Glycerate + NAD(P)^+^	Cyt, Mit, Chl	None
TSR	TSA + NAD(P)H ⇌ D-Glycerate + NAD(P)^+^	Not in plants	

* Please note that, except for HPR1, other HPRs use NADPH preferentially over NADH. Abbreviations: AHA, acetohydroxamate; AOA, aminooxyacetate; Chl, chloroplast; Cyt, cytosol; Mit—mitochondrium; OH-, hydroxy-; Per, peroxisome; Pi inorganic phosphate; SSA, succinic semialdehyde; TSA, tartronate semialdehyde; TSR, tartronate semialdehyde reductase.

## Data Availability

There are no datasets related to this study.

## Abbreviations

CBB cycle	Calvin–Benson–Bassham cycle
GABA	γ-aminobutyric acid
GHB	γ-hydroxybutyrate
GK	glycerate kinase
GR	glyoxylate reductase
HPR	hydroxypyruvate reductase
2PG	2-phosphoglycolate
3PGA	3-phosphoglycerate
PLGG1	plastidial glycolate/glycerate transporter
RA	rosmarinic acid
SGAT	serine: glyoxylate aminotransferase
SSA	succinic semialdehyde
TCA cycle	tricarboxylic acid cycle
TSR	tartronic semialdehyde reductase
VGT	vacuolar glycerate transporter
